# Critical Illness in Patients with Multiple Sclerosis: A Matched Case-Control Study

**DOI:** 10.1371/journal.pone.0155795

**Published:** 2016-05-31

**Authors:** Anush Karamyan, Martin W. Dünser, Douglas J. Wiebe, Georg Pilz, Peter Wipfler, Vaclav Chroust, Helmut F. Novak, Larissa Hauer, Eugen Trinka, Johann Sellner

**Affiliations:** 1 Department of Neurology, Christian Doppler Medical Center, Paracelsus Medical University, and Center for Cognitive Neuroscience, Salzburg, Austria; 2 Department of Anesthesiology, Perioperative and General Intensive Care Medicine, University Hospital Salzburg and Paracelsus Medical University, Salzburg, Austria; 3 Department of Biostatistics and Epidemiology, Perelman School of Medicine University of Pennsylvania, PA, United States of America; 4 Department of Psychiatry, Christian Doppler Medical Center, Paracelsus Medical University, Salzburg, Austria; 5 Department of Neurology, Klinikum rechts der Isar, Technische Universität München, Germany; Klinikum rechts der Isar der Technischen Universitaet Muenchen, GERMANY

## Abstract

**Background:**

Over the course of multiple sclerosis (MS) several conditions may arise that require critical care. We aimed to study the reasons for admission and outcome in patients with MS admitted to a neuro-intensive care unit (NICU).

**Methods:**

We retrospectively searched the electronic charts of a 9-bedded NICU in a tertiary hospital for patients with a diagnosis of multiple sclerosis (MS) from 1993–2015, and matched them to NICU controls without MS based on age and gender. Conditional logistic regression was used to compare admission causes, Charlson’s Comorbidity Index, indicators of disease severity, and survival between MS and non-MS patients.

**Results:**

We identified 61 MS patients and 181 non-MS controls. Respiratory dysfunction was the most frequent reason for NICU admission among MS patients (34.4%), having infectious context as a rule. In a matched analysis, after adjusting for co-morbidities and immunosuppressive medications, patients with MS were more likely to be admitted to the NICU because of respiratory dysfunction (OR = 7.86, 95% CI 3.02–20.42, p<0.001), non-respiratory infections (OR = 3.71, 95% CI 1.29–10.68, p = 0.02), had a higher rate of multiple NICU admissions (OR = 2.53, 95% CI 1.05–6.05, p = 0.04) than non-MS patients. Mortality after NICU admission at a median follow-up time of 1 year was higher in MS than control patients (adjusted OR = 4.21, 95% CI 1.49–11.85, p = 0.04).

**Conclusion:**

The most common reason for NICU admission in MS patients was respiratory dysfunction due to infection. Compared to non-MS patients, critically ill MS patients had a higher NICU re-admission rate, and a higher mortality.

## Introduction

Multiple sclerosis (MS) is a chronic autoimmune disease of the central nervous system, which is recognized as a leading cause of disability in working-age adults [1,2]. Immobility, involvement of vital neurological structures that may result in dysphagia and respiratory dysfunction as well as current MS medications pose a potential risk for the development of fatal complications [3,4]. While many of these conditions require critical care, the pattern of neuro-intensive care unit (NICU) admissions among MS patients has so far not been sufficiently studied. A recent study has reported a higher risk of critical care admission and 1-year mortality in MS patients as compared to the general population [5]. Given that both long-term and acute critical care of MS patients are very cost intensive [6,7], targeted interventions to reduce the need for consumption of critical care in MS patients would be paramount. To this end, it is important to better understand the reasons leading these patients to critical care presentation, as well as their outcome after admission.

The aim of this study was to describe clinical characteristics, reasons for admission and disease severity in MS patients requiring NICU admission. In addition, we compared MS patients to a matched group of NICU patients without MS. We hypothesized that critically ill MS patients would differ from non-MS patients in their admission diagnoses and mortality rate.

## Methods

### Study design and setting

This analysis was designed as a retrospective, matched case-control study. It was conducted in a 9-bed NICU of a tertiary university teaching hospital. The study protocol was reviewed and approved by the local Ethics Committee (Ethikkommision für das Bundesland Salzburg; 415-EP/73/534-2015). No written consent was needed due to the retrospective study design. Patient records were anonymized and de-identified prior to analysis.

### Study population and data collection

From January 1, 1993 until December 31, 2015, the electronic records of the NICU were reviewed for patients admitted with the diagnosis of MS, excluding those under 18 years of age or admitted for palliative care. Then, the same database was searched for non-MS patients who were matched to MS patients based on age, gender and admission year. Doing so, up to eight control subjects per MS case were identified. NICU patients admitted because of other demyelinating diseases were not considered as control matches.

The following data were extracted from the prospectively collected electronic NICU and hospital records: age (at the time of first NICU admission), gender, reason for NICU admission, use of any immunosuppressive therapy such as cyclophosphamide, methotrexate, azathioprine prior to admission (yes vs no), the Simplified Acute Physiology Score II as an indicator of disease severity during the first 24 hours after NICU admission [8], resource utilization quantified with Therapeutic Intervention Scoring System (TISS-28) [9], need for and duration of mechanical ventilation, length of NICU stay (for the initial admission), cumulative length of NICU stay (in case of re-admissions), number of NICU admissions. For each patient we retrospectively calculated the Charlson’s Comorbidity Index (CCI) as a measure of comorbidity. In MS patients, the duration of disease as well as the degree of disability at the time of NICU admission as evaluated by the Expanded Disability Status Scale (EDSS) [10], were recorded.

### Reasons for NICU admission

In MS patients, reasons for NICU admission were categorized as (1) pre-planned admissions for intensive therapeutic interventions (therapeutic plasma exchange, baclofen pump implantation); or (2) unplanned admissions due to critical illness complicating MS. In order to compare the study cohort with matched controls, reasons for admission were grouped into the following categories: respiratory dysfunction including infections; cardio-/cerebrovascular disease, neuro-/psychiatric disease, non-pulmonary infection, and planned interventions. A separate category of “other causes” included admissions for intoxication, trauma and progressive multifocal leukoencephalopathy.

### Short- and long-term mortality

The mortality status of study and control patients was determined at NICU and hospital discharge, as well as 3, 6 and 12 months after the first NICU admission, by reviewing their hospital charts until the last documentation of using medical services. We repeated the mortality analysis, excluding MS patients who were admitted to the NICU for scheduled interventions or planned drug administration, as this may have underestimated true NICU mortality.

### Statistical analysis

We report median (IQR) for continuous variables, and frequency (percent) for categorical variables. Correlations were determined using Spearman rank correlation test (r) with stratification by age, sex and CCI score.

For each study variable we used an univariate conditional logistic regression analysis to compare MS and non-MS NICU patients. To increase the power, we stratified the cohorts by 10-year age groups. We then performed multivariate conditional logistic regression analyses to control for the influence of potential confounders.

All reported p-values were two-tailed and considered statistically significant at <0.05. All statistical analyses were conducted using R statistical software version 3.2.3.

## Results

**Fig 1:**
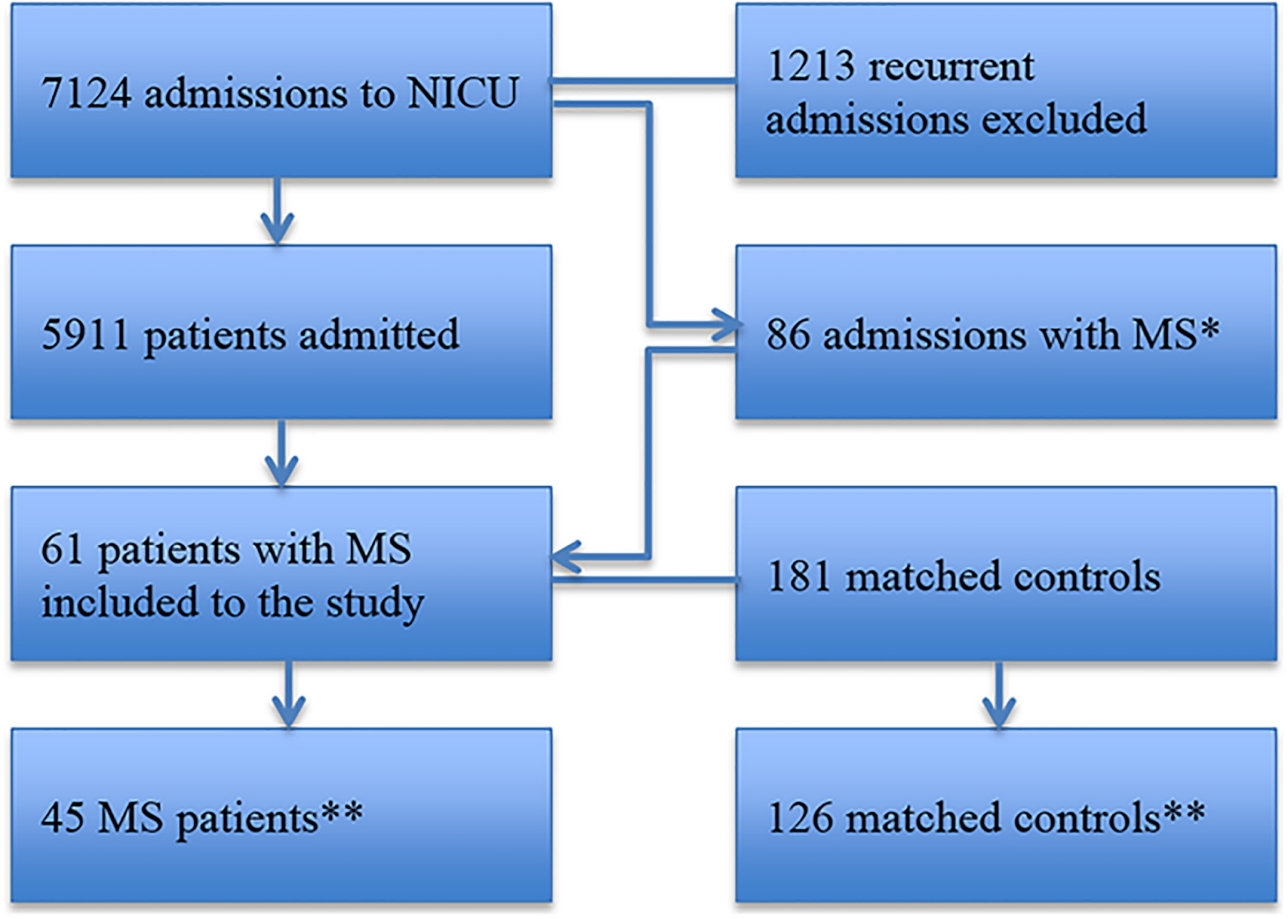
Patient population flowchart. *Admissions for plasmapheresis series (usually a total of 5 exchanges administered every other day) were considered as a single admission. **After excluding admissions for a planned intervention and adjusting the matching for mortality analysis.

Over the 22-years observation period, 7124 NICU admissions occurred, of which 5911 were primary admissions. Sixty-one patients were admitted with a diagnosis of MS (1.03%) resulting in 86 NICU admissions with a diagnosis of MS (Fig 1).

### Clinical characteristics in MS patients

Reasons for NICU admission and clinical characteristics of MS patients are summarized in Table 1. Respiratory insufficiency due to pulmonary or non-pulmonary infection was the most common reason for NICU admission (34.4%). Thirteen (21.3%) patients were admitted for plasma exchange by refractory or complicated course of the disease. MS exacerbation with infectious complications (such as decubitus) was observed in 3 (4.9%) patients, another 3 patients were admitted for MS related drug administration (e.g. cyclophospahamide, baclofen pump implantation). Less frequent causes included natalizumab associated PML (n = 3), sepsis (n = 2), trauma and severe urinary infection (n = 1). As MS patients were matched to non-MS controls based on age and gender, we compared these two parameters between MS and general non-MS NICU populations. Doing so, MS patients were younger (48 (16.7) years vs. 62 (13.7), p<0.001) and more often female (65% vs. 42%, p<0.001).

**Table 1:** Reasons for ICU admission and clinical characteristics of patients with MS.

Parameter	MS patients (n = 61)	Pre-planned admissions (n = 18)	Unplanned admissions (n = 43)	p value
Female, n (%)	39 (63.9)	10 (25.6)	29 (74.4)	0.28
Age at first admission, y	48 (29)	40.5 (17.5)	60 (20)	0.02
Age group, y, n (%)				0.02
<40	20 (33.3)	8 (40)	12 (60)	
40–59	21 (35)	9 (40.9)	13 (59.1)	
≥60	19 (31.7)	1 (5.3)	18 (94.7)	
Years since MS diagnosis	9 (13)	2 (6.8)	13 (23)	0.002
CCI, n (%)				0.07
0	26 (42.6)	11 (42.3)	15 (57.7)	
1–2	24 (39.3)	7 (26.9)	19 (73.1)	
>2	11 (18.1)	0 (0)	8 (100)	
EDSS score	4.5 (3.3)	3 (2.3)	8.5 (5.4)	0.002
Reason for admission				
Respiratory dysfunction	21 (34.4)	0 (0)	21 (100)	
Circulatory dysfunction	5 (8.2)	0 (0)	5 (100)	
Impared consciousness	5 (8.2)	2 (40)	3 (60)	
Status epilepticus	5 (8.2)	0 (0)	5 (100)	
Infection	3 (4.9)	0 (0)	3 (100)	
Plasma exchange	13 (21.3)	13 (100)	0 (0)	
Drug administration	3 (4.9)	3 (100)	0 (0)	
PML	3 (4.9)	0 (0)	3 (100)	
Sepsis	2 (3.3)	0 (0)	2 (100)	
Trauma	1 (1.6)	0 (0)	1 (100)	
SAPS II score at first admission	20 (14.8)	13 (3.5)	22.5 (27)	0.002
TISS-28 score at first admission	26 (6)	24 (3.8)	26.5 (7)	0.02
ICU length of stay at first admission, d	5 (30.6)	5 (2.5)	4 (7.8)	0.6
Readmitted patients, n (%)	13 (21.3)	3 (23.1)	10 (76.9)	0.42
Cumulative number of admissions, n	86	27	59	
ICU length of stay in total, d	5 (33.9)	5 (4)	4 (9.5)	0.7
Respiratory support, n (%)	13 (21.7)	2 (14.3)	12 (85.7)	0.14
ICU mortality, n (%)	7 (11.7)	0 (0)	7 (100)	0.009
Post-ICU mortality, n (%)	12 (20)	0 (0)	12 (100)	0.09
3-month-mortality	7 (11.7)	0 (0)	7 (100)	0.08
6-month-mortality	8 (13.3)	0 (0)	8 (100)	0.06
1-year-mortality	9 (15)	0 (0)	9 (100)	0.04

Notes: ICU, intensive care unit; MS, multiple sclerosis; EDSS, Expanded Disability Status Scale; CCI, Charlson Comorbidity Index, PML, progressive multifocal leukoencephalopathy; SAPS, Simplified Acute Physiology Score; TISS, Therapeutic Intervention Scoring System

Data shown as median (interquartile range) unless otherwise specified

Both the duration of MS history (r = 0.5, p<0.001) and the EDSS (r = 0.5, p<0.001) were associated with the SAPS II at NICU admission. The EDSS score was further associated with the length of NICU stay both for the initial admission (r = 0.32; p = 0.04), the overall length of NICU stay in case of re-admissions (r = 0.41, p = 0.008), and the number of NICU admissions (r = 0.41, p = 0.009). All associations remained significant when corrected for age, gender, the Charlson’s Comorbidity Index, and the reasons for NICU admission.

### Comparisons between MS and matched cohorts

We identified 181 matched controls in total. Relative prevalences of NICU admission reasons in both populations are shown in Table 2. MS patients had a higher relative prevalence of NICU admissions because of respiratory insufficiency, infections and therapeutic interventions. Admissions for cerebro-/cardiovascular and neuro-/psychiatric diseases, on the contrary, were less prevalent in MS patients compared to the controls. After adjusting for prior immunosuppressive treatment and comorbidities the admission rates for respiratory infections remained substantially higher in MS patients (OR = 7.86, 95% CI 3.02–20.42, p<0.001).

**Table 2:** Causes of admission, ICU characteristics and mortality in patients with MS and non-MS controls.

	Crude OR	CI 95%	p value	Adjusted OR^a^	CI 95%	p value
Causes of admission to the ICU						
Respiratory disease/infection	8.52	3.63–20	<0.001	7.86	3.02–20.42	<0.001
Cardio-/cerebrovascular disease	0.09	0.03–0.23	<0.001	0.09	0.03–0.24	<0.001
Neuro-/psychiatric disease*	0.32	0.15–0.7	0.004	0.38	0.15–0.97	0.02
Infection**	4.32	1.63–11.46	0.003	3.71	1.29–10.68	0.02
Intervention***	15.77	4.33–56.97	<0.001	8.13	2.11–31.26	0.002
Other****	0.8	0.2–3.19	0.6	0.63	0.11–3.56	0.29
CCI	1	0.99–1	0.83	n.a.	n.a.	n.a.
SAPS II	0.98	0.97–0.99	<0.001	n.a.	n.a.	n.a.
TISS-28 for the first admission	0.999	0.998–0.999	0.005	n.a.	n.a.	n.a.
ICU length of stay for first admission, d	1.01	0.99–1.03	0.32	n.a.	n.a.	n.a.
Number of readmissions, n	2.53	1.05–6.05	0.04	n.a.	n.a.	n.a.
ICU length of stay in total, d	1.02	0.99–1.03	0.13	n.a.	n.a.	n.a.
ICU mortality	4.72	1.41–15.78	0.01	4.3	1.21–15.29	<0.001
3-month mortality	3.05	1–9.31	0.049	2.83	0.85–9.58	0.15
6-month mortality	2.94	1.02–8.46	0.046	2.73	0.87–8.58	0.14
1-year mortality	3.27	1.12–9.17	0.02	3.36	1.095–10.34	0.09
Mortality over the study period	4.02	1.57–10.35	0.004	4.21	1.49–11.85	0.04

Notes: OR—odds ratio, CI—confidence interval, CCI—Charlson comorbidity index, SAPS—simplified acute physiology score, TISS—therapeutic intervention scoring system, n.a.—non applicable

*Status epilepticus, Guillain-Barré syndrome, polyneuropathy, generalized tetanus, encephalitis

**Urinary infection, sepsis, decubitus; except respiratory infections

***Plasma exchange, drug administration, coiling

****Intoxication, abscess, trauma, progressive multifocal leukoencephalopathy

^a^Calculated using a multivariate model incorporating the use of immunesuppressive medication and Charlson score

### Mortality analyses

The median follow-up time for MS and matched controls was one year. In MS patients, mortality at NICU discharge, hospital discharge, 3 months, 6 months and 1 year following NICU admission was 11.5%, 11.5%, 11.5%, 13.1%, and 14.8%, respectively. The mortality rate during the observation period was 20%. In the age and gender stratified conditional regression analyses controlling for comorbidity index, MS patients had a 5.6-fold (95% CI 1.2–25.6, p = 0.03) increased risk of dying in the NICU compared to non-MS patients. When we stratified the cohorts by age in 10-year increments, the mortality remained higher in the MS cohort. In a multivariate model only the number of NICU re-admissions proved to be an independent predictor of NICU mortality (OR = 2.7, 95% CI 1.2–6.3, p = 0.02) in MS patients, while the Charlson’s Comorbidity Index, reasons for admission causes, SAPS II and prior immunosuppressive therapy were not (Table 2). Including the variable indicating years of admission for each patient into the model did not influence the results.

## Discussion

To the best of our knowledge, this is the first hospital-based study focusing on NICU admissions and outcome of MS patients. Our results indicate that MS patients were admitted to the NICU mainly because of respiratory insufficiency related to infections. The need for medical interventions such as therapeutic plasma exchange, baclofen pump implantation was another common reason for NICU admission in this population, and is observed to a much lesser extent in the remaining ICU population. Infections of non-respiratory nature, taken separately, are more frequent in the MS population compared to demographically similar ICU population without MS, as well. Whatever the cause of admission, the MS population is younger on average than the overall ICU population, and has higher ICU, 1-year, as well as the study period mortality adjusted for comorbid state.

Prior work on critically ill patients with MS is sparse. Our findings are consistent with the report from Marrie et al. suggesting that infections constitute the most common cause for ICU admissions in MS patients [4]. In their population-based study they estimated 1.7-fold adjusted OR (95% CI 1.01–2.90) for the risk of admission for infection in the MS compared to the general population. Notably, a Swedish study reported an adjusted relative risk of 4.26 (95% CI 4.13–4.40) for MS patients vs. general population to be admitted to the hospital due to infections [11]. In their study on autoimmune diseases in the ICU Bernal-Macias et al. observed the main causes of admission to be infections and disease flare up (36% and 24%, respectively) [12]. In our cohort, the admissions for only respiratory infections were 7.86-fold (95% CI 3.02–20.42, p<0.001), and for other infections 3.71-fold (95% CI 1.29–10.68, p = 0.02) more frequent, as compared to the general ICU population. In this setting the finding has not been previously reported. Nevertheless, the literature suggests frequent occurrence of respiratory dysfunction in patients with MS, particularly in advanced stages of the disease [13].

Our cohort was demographically different from the entire ICU population having particularly younger age at admission, and consisting predominantly of women. The latter might be the reflection of the increasingly high female to male ratio in the MS population [14, 15]. On the other hand, the literature provides several reports on male predominance among the ICU population [16], and mean age of 62.3±17.6 years [17], similar to the demographic pattern of our ICU.

The positive correlations between the MS duration and ICU scores allow us to assume that patients with MS become susceptible to more severe critical illness in later stages of the disease. The SAPS II score, which is designed to predict mortality in critically ill patients, correlated with longer MS duration, suggesting that patients admitted to the ICU with longer prior history of MS appear to be more severely ill, as assessed by SAPS II scores. A study at one large NICU found TISS-28 scores at admission to be independent predictors of unfavorable outcome in a neurocritical care population as well [18]. Interestingly, TISS-28 and SAPS II scores did not correlate with mortality in our MS cohort. Therefore we suppose that the instruments might be not enough accurate for the MS population. Whatever the case, validation studies are necessary.

A number of studies have reported higher mortality rates in MS patients compared to the general population [19,20,21]. In a population-based cohort study Lalmohamed et al. estimated a 3,5-fold increased all-cause mortality rate among patients with MS, compared with referent subjects, and particularly the highest mortality hazard ratio (HR) for infectious/respiratory-related deaths (HR 7.69, 95% CI 4.92–12.02) [21]. Furthermore, there are reports on increased mortality risk of ICU admitted patients in the subsequent years after discharge as compared with the general population [22]. In current study we estimated the mortality in the ICU admitted MS cohort to be higher than in the ICU population. In addition, we found an increased risk of readmissions in the MS cohort, which was an independent predictor of ICU mortality among them. Similarly, in their multicenter cohort study Metnitz and colleagues describe an ICU subpopulation with higher rate of readmissions, which had a fourfold risk of hospital mortality [23].

Our work has several limitations. As a retrospective cohort study it might be subject to various unknown confounders because of the lack of necessary adjustments. Hospital-based data derived from a single center, and the relatively small sample size, on the other hand, might carry a potential bias. However, the center is unique in its nature serving a large region of the country. We performed a matched analysis within the admissions of each year separately to minimize the impact of temporal changes in management and outcomes. We did not perform subgroup analyses within the MS cohort due to the limited sample size. Nevertheless, despite these limitations, our study provides clear evidence of unfavorable short- and long-term outcome of MS patients admitted to the ICU. Further larger multicenter studies are required to confirm our findings.

## Conclusions

MS patients comprise a non-negligible subpopulation in the ICU with specific demographic and clinical characteristics that differ from the overall ICU population. Even though these patients make up a relatively small proportion in the ICU, but are prominent with their younger age at admission, higher mortality despite relatively less severe chronic status and higher re-admission rate. Therefore increased alertness of healthcare providers towards this population and reduction of potentially preventable conditions leading to severe illness such as respiratory infections is necessary.
